# Unique Allelic eQTL Clusters in Human MHC Haplotypes

**DOI:** 10.1534/g3.117.043828

**Published:** 2017-06-09

**Authors:** Tze Hau Lam, Meixin Shen, Matthew Zirui Tay, Ee Chee Ren

**Affiliations:** * Singapore Immunology Network, A*STAR, Singapore 138648; † Department of Molecular Genetics and Microbiology, Duke University, Durham, North Carolina 27710; ‡ Department of Microbiology and Immunology, Yong Loo Lin School of Medicine, National University of Singapore, Singapore 117597

**Keywords:** MHC, haplotypes, MHC sequence polymorphism, eQTLs

## Abstract

The control of gene regulation within the major histocompatibility complex (MHC) remains poorly understood, despite several expression quantitative trait loci (eQTL) studies revealing an association of MHC gene expression with independent tag-single nucleotide polymorphisms (SNPs). MHC haplotype variation may exert a greater effect on gene expression phenotype than specific single variants. To explore the effect of MHC haplotype sequence diversity on gene expression phenotypes across the MHC, we examined the MHC transcriptomic landscape at haplotype-specific resolution for three prominent MHC haplotypes (A2-B46-DR9, A33-B58-DR3, and A1-B8-DR3) derived from MHC-homozygous B-lymphoblastoid cell lines (B-LCLs). We demonstrate that MHC-wide gene expression patterns are dictated by underlying haplotypes, and identify 36 differentially expressed genes. By mapping these haplotype sequence variations to known eQTL, we provide evidence that unique allelic combinations of eQTL, embedded within haplotypes, are correlated with the level of expression of 17 genes. Interestingly, the influence of haplotype sequence on gene expression is not homogenous across the MHC. We show that haplotype sequence polymorphisms within or proximate to HLA-A, HLA-C, C4A, and HLA-DRB regions exert haplotype-specific gene regulatory effects, whereas the expression of genes in other parts of the MHC region are not affected by the haplotype sequence. Overall, we demonstrate that MHC haplotype sequence diversity can impact phenotypic outcome via the alteration of transcriptional variability, indicating that a haplotype-based approach is fundamental for the assessment of trait associations in the MHC.

Genome-wide association studies (GWAS) have identified thousands of SNPs that confer association with >1600 observable phenotypes, complex traits, and diseases (Welter *et al.* 2014). As a result of the massive number of possible individual SNPs in the human genome, tag-SNPs, which are representative of a short sequence block or haplotype held together by linkage disequilibrium, are used to define a genomic block (International HapMap Consortium 2003). Notably, the majority (>90%) of these tag-SNPs are found in noncoding regions of the human genome (Maurano *et al.* 2012), presenting a major challenge for our understanding of the mechanistic effects of these variants on common traits. eQTL mapping of DNA sequence polymorphisms that modify transcript abundance of gene(s) has since emerged as an effective approach to reveal the implication of trait-associated variants on observable phenotypes (Albert and Kruglyak 2015). This approach has provided compelling evidence for eQTL causality in numerous traits, such as neurological disorders, autoimmune disorders, and cancers (Fairfax *et al.* 2014; Li *et al.* 2014; Ongen *et al.* 2014; Raj *et al.* 2014; Ramasamy *et al.* 2014).

The MHC region is one of the most prominent and intensely studied regions in the entire human genome. The genomic architecture of the MHC region displays high gene density, extreme sequence polymorphism, and high linkage disequilibrium (Horton *et al.* 2004). More importantly, GWAS have consistently implicated tag-SNPs located within the MHC region with a range of traits (Trowsdale and Knight 2013). Although several eQTL studies have revealed the control of MHC gene expression by single independent tag-SNPs (Vandiedonck *et al.* 2011; de Jong *et al.* 2012; Fairfax *et al.* 2012), functional variants implicating trait risks within the MHC have not been fully determined. Tag-SNPs are described as being embedded within specific haplotypes, which include functional variants that confer trait susceptibility (Balding 2006; Frazer *et al.* 2007). However, the highly polymorphic nature of the MHC coupled with extensive linkage disequilibrium gives rise to vast haplotype diversity of varying length that cannot be fully reflected by tag-SNPs (de Bakker *et al.* 2006; Gourraud *et al.* 2014). In addition, multiple loci within the MHC can contribute to disease development, and are often in tight linkage disequilibrium with one another (Feng *et al.* 2009; Rioux *et al.* 2009). As the result of these factors, haplotype variation in the MHC exerts a greater effect on phenotypic outcomes than a specific single variant (Vandiedonck and Knight 2009). Indeed, numerous MHC haplotypes that are found in relatively high incidence in the general population are frequently implicated in various autoimmune disorders (Traherne *et al.* 2006). One example is the A1-B8-DR3 MHC haplotype that is associated with type 1 diabetes, systemic lupus erythematosus, IgA deficiency, and celiac disease (Schroeder *et al.* 1998; Price *et al.* 1999). Yet, when compared with the causality of single variants to traits, our current understanding of how haplotype combinations affect phenotypic consequences is inadequate. A key component for understanding this complex relationship is through the analysis of MHC haplotype sequence variations (de Bakker and Raychaudhuri 2012; Larsen *et al.* 2014). As such, the ability to demonstrate how MHC haplotype sequence variability can modify gene transcript abundance within the MHC will be an important step toward improving our understanding of the biological effects of specific MHC haplotypes.

To understand the effect of MHC haplotype sequence diversity on gene expression phenotype, we conducted an integrated analysis using three genetic approaches: (a) characterization of MHC transcriptomics derived from RNA sequencing (RNA-Seq) of MHC-homozygous B-LCLs, (b) analysis of multiple MHC haplotype sequences and their polymorphisms at nucleotide resolution, and (c) mapping of known eQTL to these haplotype-specific polymorphisms. This approach allowed us to differentiate eQTL that are fixed within haplotypes, and enabled accurate mapping of potential regions and variants that impact gene regulation within the MHC. Through this integrated analysis, we demonstrate a correlation between eQTL and MHC haplotype sequence variation, and identify haplotype-specific eQTL combinations that exert control over gene expression within the MHC region.

## Materials and Methods

### Cell lines selection

Eight B-LCLs that are homozygous at the HLA-A, -B, -C, and DRB1 loci, were selected for RNA-Seq. Another 31 B-LCLs with at least one copy of their chromosomes including either the A2-B46-DR9 (*n* = 15) or A33-B58-DR3 (*n* = 16) haplotype were used to validate expression data from RNA-Seq. All cells were prepared and provided by the Singapore Immunology Network, A*STAR, Singapore and the Research Cell Bank and Fred Hutchinson Research Centre, Seattle, WA. HLA loci were typed using a sequence-based approach as described previously (Lam *et al.* 2015). B-LCL cultures were stimulated for 6 hr at 37° with 200 nM of phorbol 12-myristate 13-acetate (Sigma) and 125 nM of ionomycin to activate the cells.

### Quantitative real-time PCR (qPCR)

RNA extraction was performed using an RNeasy Mini kit (QIAGEN) according to the manufacturer’s instructions, and RNA was quantitated using a NanoDrop ND-1000 spectrophotometer (Thermo Fisher Scientific). Samples (100 ng/μl) were transcribed into cDNA using the AccuScript High Fidelity 1st Strand cDNA Synthesis Kit (Agilent). Gene-specific qPCR was performed for triplicate samples on the Applied Biosystems 7500 Real-Time PCR System using the KAPA SYBR FAST qPCR Master Mix (KAPA Biosystems). Gene-specific primers were designed using NCBI Primer-Blast. Primer sequences are available in Supplemental Material, Table S1. Relative quantities of RNA were measured using ΔΔ*CT* normalized to HPRT levels. Differential gene expression between haplotypes was assessed using the Mann–Whitney *U* test.

### RNA-Seq

The quality and concentration of total RNA, extracted using the RNeasy Mini Kit (QIAGEN), was measured using an Agilent Bioanalyzer 2100. Only samples with an RNA integrity number of >7.0 were selected for RNA-Seq. Prior to library preparation, ribosomal RNA was removed from samples using the human Ribo-Zero rRNA Removal Kit (Epicentre). Paired-end RNA-Seq libraries were generated using the Illumina TruSeq RNA library preparation kit v2 (Illumina, San Diego, CA). A total of 16 libraries were prepared (two libraries per cell line) and sequenced on the Illumina HiSeq 2000 machine at two libraries per lane.

### Processing of RNA-Seq reads and expression analysis

Raw sequencing reads with 70% of base positions having a Phred score of <20 were removed prior to alignment using the NGS QC Toolkit (Patel and Jain 2012). The processed reads were then mapped to the hg19 human genome assembly using TopHat2 v2.0.13 (Trapnell *et al.* 2009). Reads mapping to genomic features in the GENCODE release 19 (GRCh37.p13) gene annotation were summarized using the featureCounts program in the Subread package v1.5.0-p1 (Liao *et al.* 2014). For a read pair to be assigned to a genomic feature, it has to be uniquely mapped to a single genomic location, both ends must be concordantly mapped, and it should have a mapping quality score ≥ 25. The DESeq2 v1.10.1 (Love *et al.* 2014) was used to normalize the RNA-Seq libraries, and limma v3.26.8 (Ritchie *et al.* 2015) was used to perform differential expression evaluation. Only loci with a sum of normalized read counts across all samples >10 were considered as expressed, and genomic regions mapping to multiple genomic features were not considered for differential expression analysis. The Benjamini and Hochberg method was used to control for multiple testing.

### MHC haplotype sequence and data analysis

The Chinese A2-B46-DR9 and A33-B58-DR3 haplotype sequences were derived from Lam *et al.* (2015), and the BED file containing sequencing information for the A1-B8-DR3 haplotype was downloaded from https://www.ucl.ac.uk/cancer/research/department-cancer-biology/medical-genomics-group/past-projects/mhc-haplotype-project. The coordinates of the haplotype sequence were based on the Human Reference Sequence Assembly 37.2 (NCBI build 37.2). Haplotype sequence comparisons were performed using in-house written R-scripts (Lam *et al.* 2015).

*Cis*-acting eQTL SNPs specific to transformed B-LCLs isolated from healthy individuals were obtained from the Genotype-Tissue Expression (GTEx) consortium (Genotype-Tissue Expression Project 2013) database (Release V6). Only SNPs with an adjusted P-value < 0.05 were considered for analysis. Regularized log transformation, implemented by DESeq2, was applied to normalized raw counts, and was then used as input for hierarchical clustering and principal component analysis (PCA). Hierarchical clustering, PCA, k-means clustering, and calculation of Cohen’s κ coefficient were all implemented using packages in R.

### Data availability

The gene expression data are available at GEO using the accession number GSE83403.

## Results

### MHC-wide gene expression patterns

The correlation of MHC-wide gene expression profiles with MHC haplotypes was examined by transcriptome sequencing of eight EBV-transformed B-LCLs that are HLA homozygous at the HLA-A, -B, -C, and DRB1 loci. Of the eight cell lines tested, three (B58AL, B58SC, and B58CF) carry the Chinese A*33:03-C*03:02-B*58:01-DRB1*03:01 (A33-B58-DR3) MHC haplotype; three (B46BM, B46ZS, and B46CM) carry the Chinese A*02:07-C*01:02-B*46:01-DRB1*09:01 (A2-B46-DR9) MHC haplotype; and two (ARC and COX) carry the European A*01:01-C*07:01-B*08:01-DRB1*03:01 (A1-B8-DR3) MHC haplotype. The sequences of these three MHC haplotypes were previously established and are found to exhibit features of conserved extended haplotypes (CEHs), where long stretches of dominant sequence across the entire MHC genomic region remain intact (Stewart *et al.* 2004; Aly *et al.* 2006; Lam *et al.* 2015). The use of HLA homozygous cell lines of MHC haplotypes that include CEHs ensures that both copies of the chromosomes at the MHC region are representative of the haplotype of interest, and should provide an accurate depiction of the MHC region’s transcriptome profile of a particular MHC haplotype.

**Figure 1:**
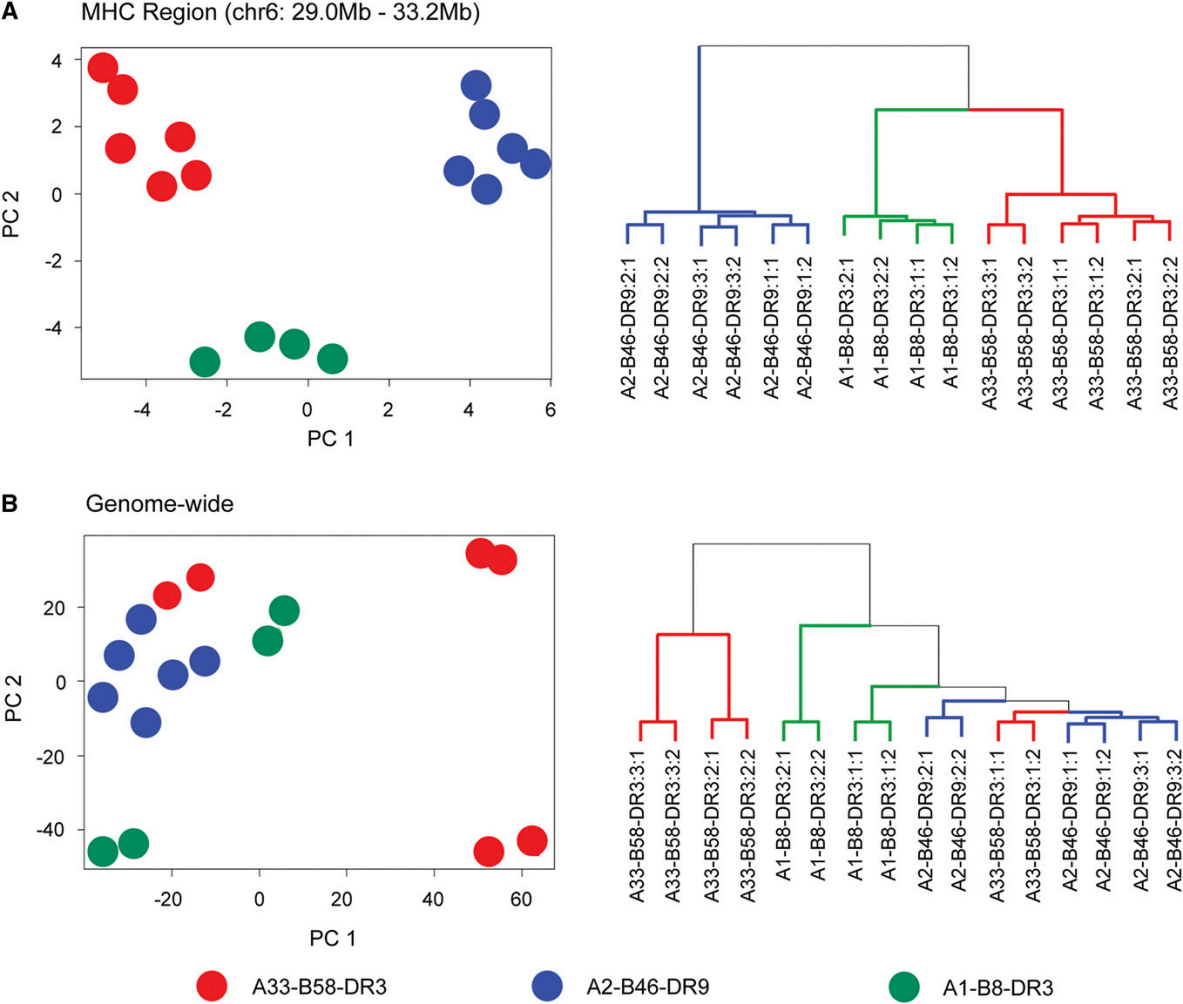
MHC-wide gene expression patterns defined by MHC haplotypes. (A) Hierarchical and PC analysis based on expression of 177 MHC-residing loci found within chr6:29.0–33.2 Mb. (B) Hierarchical and PC analysis based on expression of genes in the human genome. Expression profiles were derived from RNA-sequencing of eight B-LCLs with two libraries generated per B-LCL. Blue indicates B-LCLs carrying the A2-B46-DR9 haplotype, red indicates B-LCLs carrying the A33-B58-DR3 haplotype, and green indicates B-LCLs carrying the A1-B8-DR3 haplotype. B-LCL, B-lymphoblastoid cell line; chr, chromosome; MHC, major histocompatibility complex; PC, principal component.

We analyzed the expression of genes residing in the MHC genomic region from chr6:29.0–33.2 Mb. Under the GENCODE (GRCh37.p13) gene annotation, there are a total of 338 loci (inclusive of protein-coding, pseudogene, and noncoding RNAs) found within the MHC region, and 177 of these were found to be expressed in the EBV-transformed B-LCLs. To examine the transcriptome profiles of the genes expressed within the MHC region, regularized log values of the 177 loci were fitted for hierarchical clustering and PCA. From the analysis plots, we observed that samples carrying identical MHC haplotypes were distinctly clustered together (Figure 1A). In contrast, when a similar analysis was performed using the whole-genome transcriptomic data, no detectable cluster was observed (Figure 1B). We then performed a more refined comparison by analyzing the gene expression pattern within chromosome 1 as well as chromosome 6 (excluding the genes in the MHC region) as controls and again no detectable cluster was seen (Figure S1 in File S1). These results indicate that the clustering pattern derived from gene expression is exclusive to the MHC region, suggesting that MHC-wide gene expression patterns are associated with the underlying MHC haplotype carried by each individual.

### Genes differentially expressed in the MHC region

**Figure 2:**
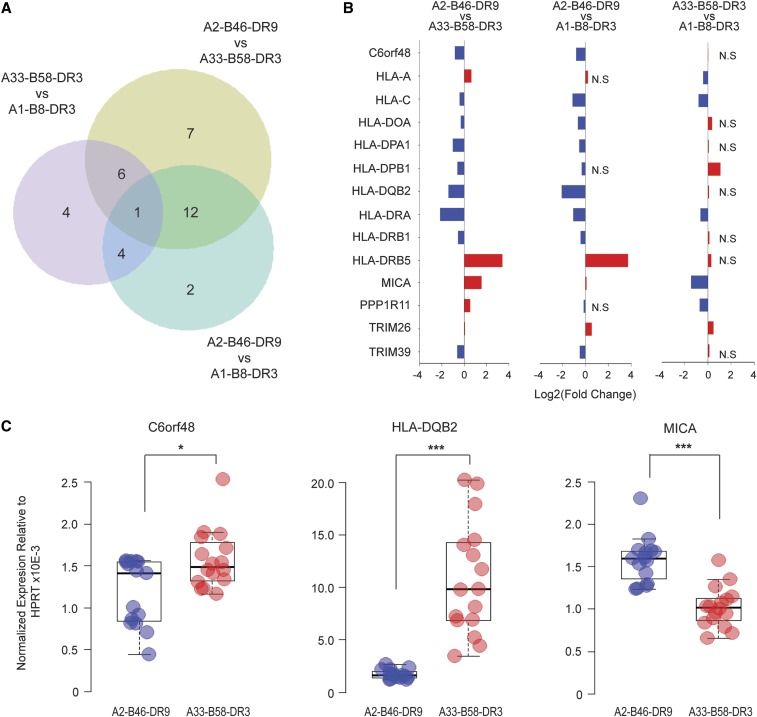
Differential gene expression analysis between haplotypes. (A) Venn diagram indicating the number of significant differentially expressed genes (adjusted P-value < 0.05) between pairwise haplotype comparisons. (B) Top 15 differentially expressed genes with adjusted P-value < 0.001 in at least one of the pairwise haplotype comparisons. (C) Relative expression of C6orf48, HLA-DQB2, and MICA between the A2-B46-DR9 (*n* = 15) and A33-B58-DR3 haplotypes (*n* = 16) in B-LCLs (Mann–Whitney *U* test). * P-value < 0.05, *** P-value < 0.0001. HPRT, hypoxanthine phosphoribosyltransferase.

Using the MHC haplotype transcriptome profiles generated from RNA-Seq, we proceeded to investigate MHC haplotype-specific gene expression. We identified 36 significantly differentially expressed genes across the three MHC haplotypes (adjusted P-value < 0.05) (Table S2); 27 genes between A2-B46-DR9 and A33-B58-DR3; 20 genes between A2-B46-DR9 and A1-B8-DR3; as well as 16 genes between A33-B58-DR3 and A1-B8-DR3 (Figure 2A). Of these genes, only the HLA-DRA gene was found to be differentially expressed in all three pairwise haplotype comparisons. From the examination of the top differentially expressed genes (adjusted P-value < 0.001), we observed that 9 out of 14 were classical HLA class I and class II genes (Figure 2B), suggesting that haplotype sequence variations have a greater impact on the level of the HLA gene expression than other loci residing within the MHC region. To validate the RNA-Seq results, we selected an independent cohort of B-LCLs where at least one copy of their chromosome has either the A2-B46-DR9 or A33-B58-DR3 haplotype, and quantified the level of expression of C6orf48, HLA-DQB2, and MICA in these B-LCLs using real-time PCR. The results obtained correlated with the trend observed by RNA-Seq, providing further evidence for MHC haplotype-specific transcriptomic signatures (Figure 2C).

### MHC haplotype sequence variation controls gene expression

The presence of haplotype-specific differentially expressed genes observed in our study suggests an effect of haplotype sequence polymorphisms on gene regulation within the MHC region. To further examine this possible relationship, we first acquired *cis*-acting eQTL SNPs specific to EBV-transformed B-LCLs from the GTEx project (Carithers and Moore 2015), which are retained eQTL SNPs with a false discovery value < 0.05 and are reported to be associated with haplotype-specific differentially expressed genes. Of the 36 haplotype-specific differentially expressed genes identified above, 17 genes have reported eQTL SNPs that control their expression. Next, we obtained the haplotype sequence (chr6:29.0–33.2 Mb) representative of A2-B46-DR9, A33-B58-DR3, and A1-B8-DR3 established in published studies (Stewart *et al.* 2004; Traherne *et al.* 2006; Lam *et al.* 2015), and performed pairwise haplotype sequence comparisons. We then mapped the haplotype sequence variations to the eQTL SNPs that influenced the expression of the 17 identified differentially expressed genes (Table S3), and we assessed these eQTL with the corresponding gene expression of the specific haplotype.

**Figure 3:**
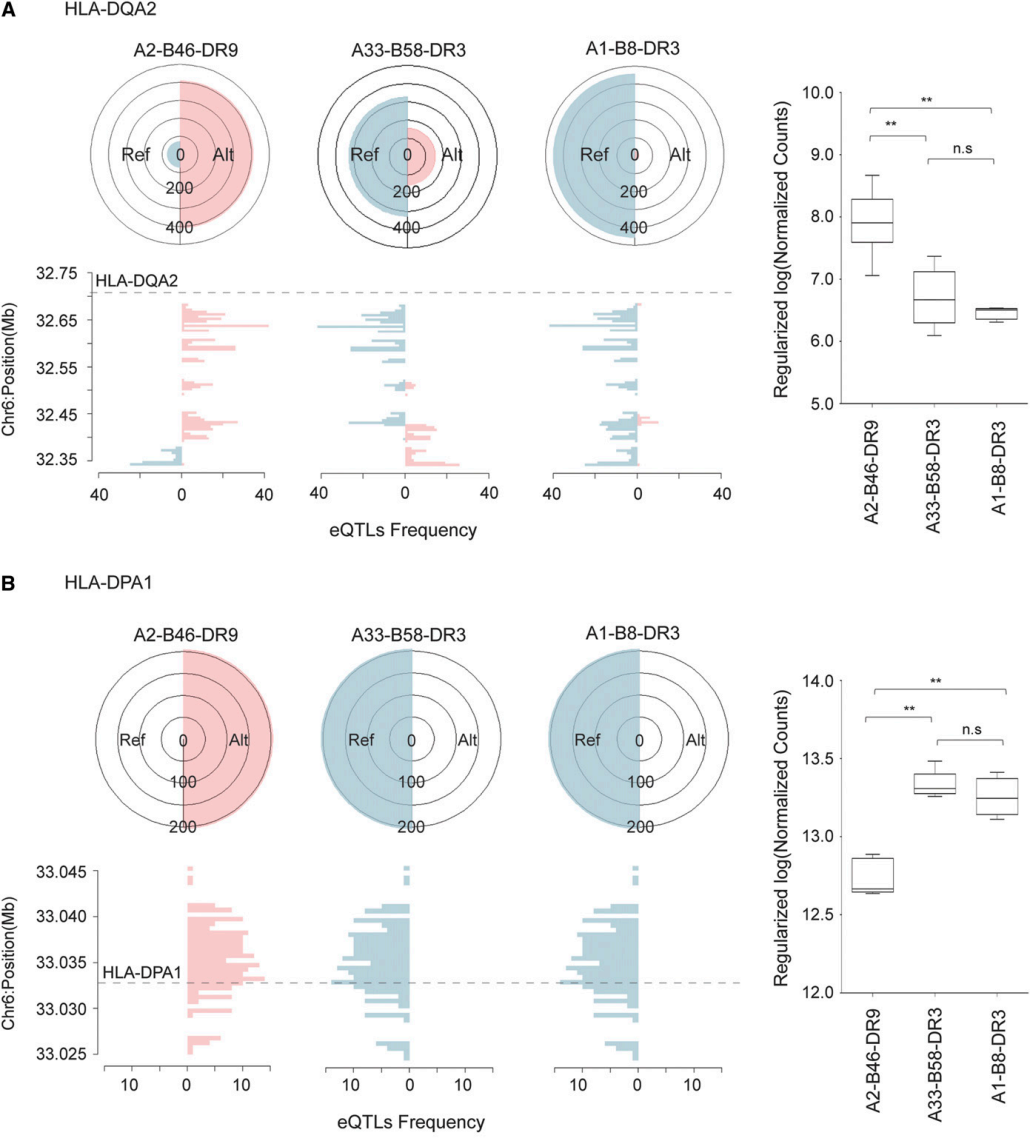
Allelic proportions of the haplotype sequence variations mapping to eQTL SNPs and the expression level of the target gene for (A) HLA-DQA2 and (B) HLA-DPA1. In each panel, the top left side shows the number of eQTL SNPs with reference allele (blue) and alternate allele (pink) for each haplotype while the bottom left side shows the frequency of eQTL at each 5 kb window relative to the location of their target gene. The right side of each panel presents the gene expression level for each haplotype derived from eight MHC-homozygous B-LCLs. ** Adjusted P-value < 0.05. B-LCL, B-lymphoblastoid cell line; eQTL, expression quantitative trait loci; MHC, major histocompatibility complex; n.s, not significant; SNP, single nucleotide polymorphism.

We identified 481 haplotype sequence variations mapping to eQTL SNPs where the alternate alleles are associated with an upregulation of HLA-DQA2 gene expression. In the A2-B46-DR9 haplotype, 409/481 of these eQTL SNPs possess the alternate allele, while in the A33-B58-DR3 and A1-B8-DR3 haplotypes, 155/481 and 24/481 eQTL SNPs possess the alternate allele, respectively (Figure 3A). When we examined HLA-DQA2 expression, we found that the A2-B46-DR9 haplotype, which exhibits the highest proportion of alternate allele eQTL SNPs, also has significantly higher gene expression compared with the other haplotypes. We identified 204 haplotype sequence variations mapping to eQTL SNPs where the reference alleles are associated with an upregulation of HLA-DPA1 expression (Figure 3B). In this case, the A33-B58-DR3 and A1-B8-DR3 haplotypes exhibit the reference allele for 100% (*n* = 204) of the eQTL SNPs compared to 0% in the A2-B46-DR9 haplotype. Subsequently, we determined that HLA-DPA1 expression is significantly higher in both the A33-B58-DR3 and A1-B8-DR3 haplotypes than in the A2-B46-DR9 haplotype (Figure 2B).

**Figure 4:**
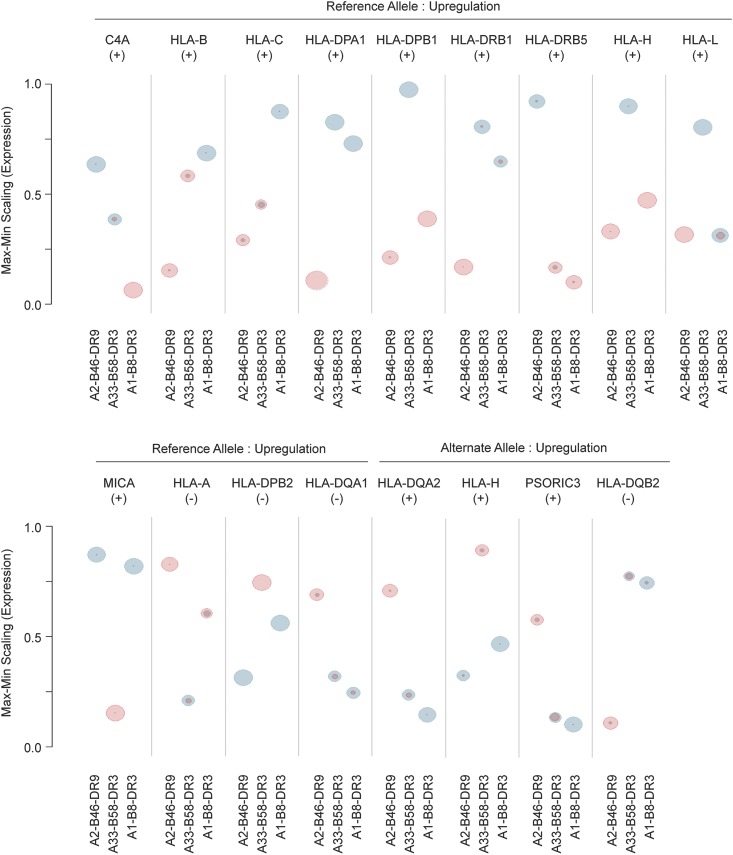
Correlation of allelic proportion of eQTL to expression levels of genes that are differentially expressed between haplotypes. The expression of genes was rescaled relatively among the haplotypes. The size of the spheres represents the relative number of eQTL with reference allele (blue) or alternate allele (pink) for each haplotype. The (+)/(−) notations below each gene name indicates the direction of the correlation for each corresponding gene. The exact count of the allelic proportion of the eQTL for each gene is shown in Figure S2 in File S1. eQTL, expression quantitative trait loci; Max, maximum; Min, minimum.

To identify if haplotype sequence variations at eQTL SNPs corresponded to gene expression for the 17 differentially regulated genes identified, we rescaled the expression of the 17 genes for evaluation among the three haplotypes and determined the allelic proportion of the haplotype sequence variations mapping to eQTL SNPs (Figure S2 in File S1). All 17 genes with reported eQTL information showed significant correlations between their expression and the allelic proportion of their haplotype sequence variations, with 13 correlating positively and 4 correlating inversely (Figure 4). Thus, we provide evidence of haplotype sequence variations that function as *cis*-acting regulatory variants, controlling expression levels of multiple genes across the MHC region.

### eQTL allelic combinations embedded in long-range MHC haplotypes

**Figure 5:**
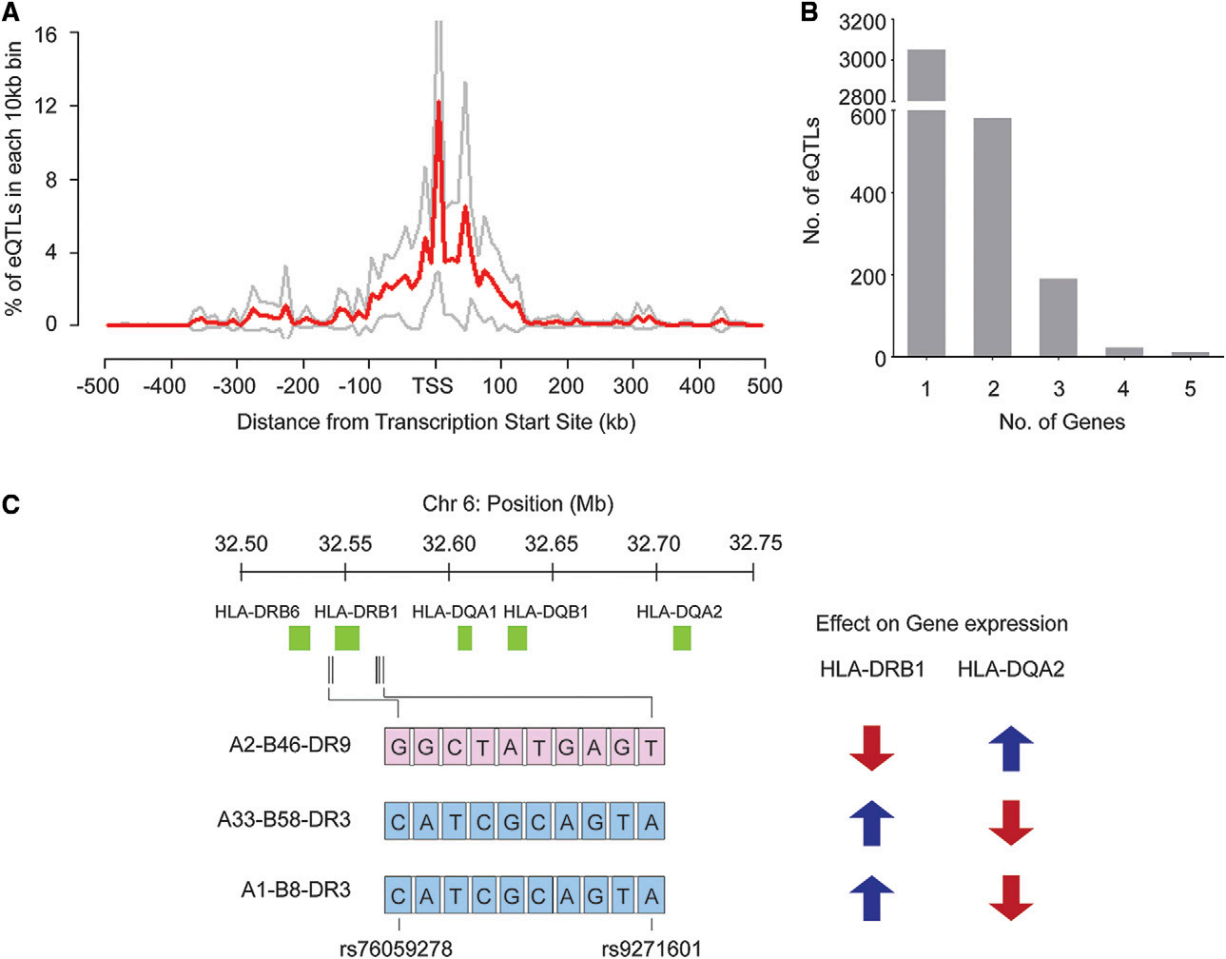
Haplotype-specific eQTL allelic combinations. (A) Percentage of eQTL in each 10 kb bin from the gene transcription start site. Red line denotes the average percentage for the 17 differentially expressed genes. Gray lines denote the upper and lower bounds of the 95% C.I. (B) Bar charts illustrating the quantity of target genes regulated by eQTL. (C) Bidirectional expression level of HLA-DRB1 and HLA-DQA2 regulated by haplotype-specific eQTL allelic combinations. The A2-B46-DR9 haplotype holds the alternate allelic sequence (pink), and the A33-B58-DR3 and A1-B8-DR3 haplotypes hold the reference allelic sequence (blue) at the 10 eQTL sites. Chr, chromosome; eQTL, expression quantitative trait loci.

Importantly, our study finds that multiple regulatory sites are fixed in specific allelic combinations and are embedded in the MHC haplotype to direct gene expression. On average, these haplotype-specific allelic combinations consist of 297 (median) eQTL sites where the majority are located within 200 kb of their target genes (Figure 5A). Genes such as C4A, HLA-DRB5, and HLA-DQA2 had >400 eQTL in linkage disequilibrium, extending to >350 kb. In total, 3857 eQTL were mapped to haplotype sequence variations that regulate the 17 identified differentially expressed genes. Interestingly, 805 of these sites were found to affect expression of >1 gene locus (Figure 5B), where 97.8% (787/805) of these sites were clustered to regions proximate to the HLA-C and HLA-DR regions; sites that regulated >2 genes were found exclusively in the HLA-DRB region (Figure S3 in File S1). Detailed analysis of the eQTL allelic sequence revealed haplotype-specific allelic combinations consisting of 10 eQTL covering 87 kb, which are associated with bidirectional expression levels of HLA-DRB1 and HLA-DQA2. The alternate allelic sequence of these 10 eQTL, carried by the A2-B46-DR9 haplotype, was associated with downregulation of HLA-DRB1 and upregulation of HLA-DQA2 expression, while the reference allelic sequence, embedded in the A33-B58-DR3 and A1-B8-DR3 haplotypes, displayed a reverse relationship (Figure 5C). Overall, the occurrence of multiple eQTL existing in specific allelic combinations embedded within an MHC haplotype suggests that these eQTL could cooperatively influence the expression of nearby genes.

### Localized regions dictate MHC-wide expression patterns

**Figure 6:**
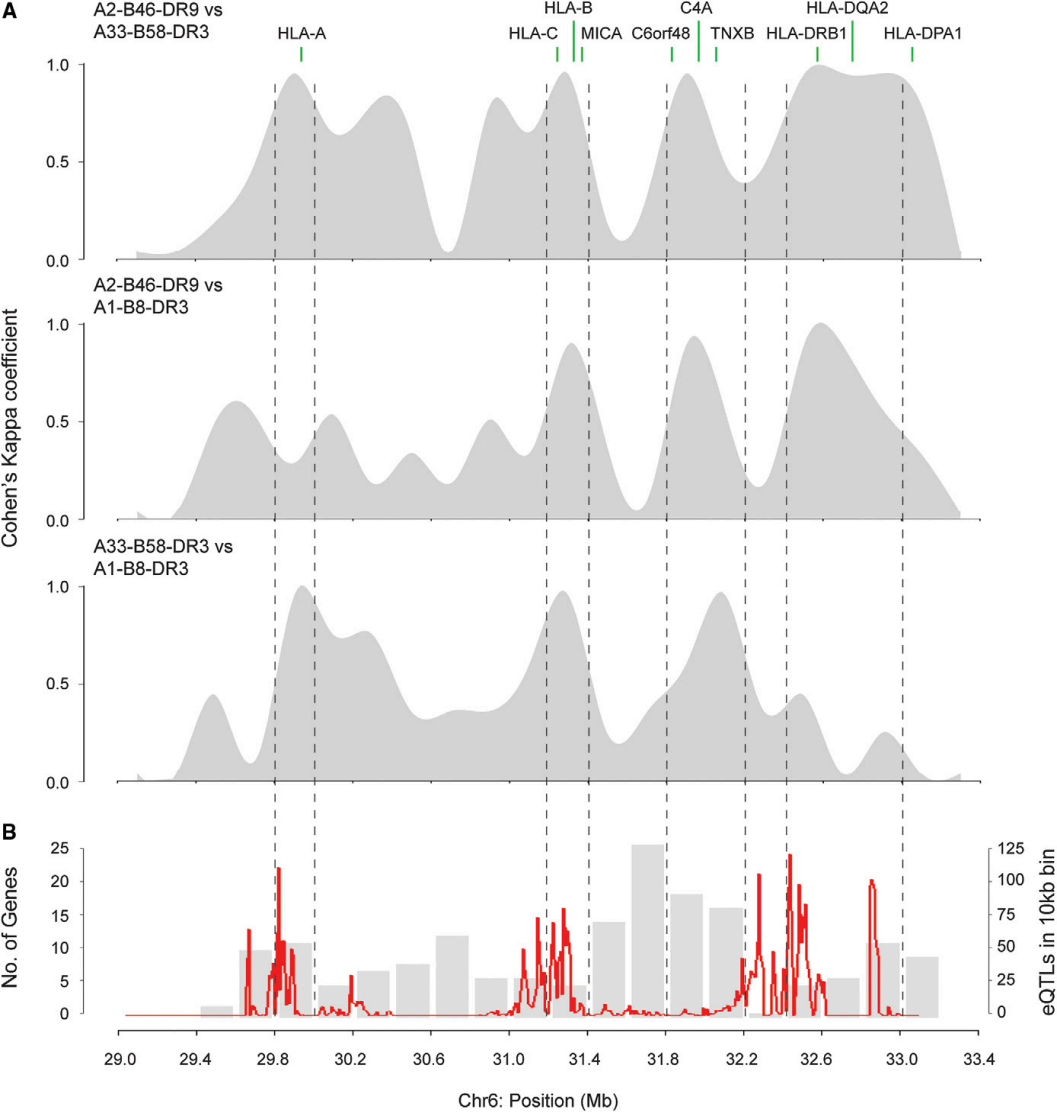
Regions that define MHC-wide expression patterns. (A) Pairwise haplotype comparisons of the agreement of 200kb window cluster patterns with MHC-wide cluster patterns. (B) Regions within the MHC region enriched with eQTL that dictate the transcription variability of the haplotype-specific differentially expressed genes (red). Gray bars indicate gene density in each 200-kb window. Chr, chromosome; eQTL, expression quantitative trait loci; MHC, major histocompatibility complex.

To identify the major contributory regions within the MHC toward the observed haplotype-specific gene expression pattern, we binned chr6:29.0–33.2 Mb into windows of 200 kb and performed a cluster analysis based on the expression of genes located in each of the 200 kb windows. We then assessed the agreement of the 200 kb regional clustering result with the MHC-wide clustering pattern from the pairwise haplotypes comparison using Cohen’s κ coefficient metrics. We observed that gene expression clustering in four of the localized regions (29.8–30.0, 31.2–31.4, 31.8–32.2, and 32.4–33.0 Mb) corresponded with the MHC-wide cluster pattern (Figure 6A) and, with the exception of the 31.8–32.2 Mb region, three of these encompass HLA genes. In addition, the enrichment of haplotype-specific eQTL either within or proximate to these regions (Figure 6B) suggests that haplotype sequence variation in these regions is a possible contributory determinant in defining gene expression within the MHC region. Notably, the gene expression pattern in the HLA-DRB region for the A33-B58-DR3 and A1-B8-DR3 haplotypes cannot be differentiated by MHC haplotype. These haplotypes both carry the HLA-DRB1*03:01 allele and share almost identical sequence at the HLA-DRB region (Lam *et al.* 2015). This corresponds with our observation that sequence similarity results in similar levels of gene expression between the two haplotypes. Together, this data highlights the importance of MHC haplotype variation on gene expression.

## Discussion

We examined the MHC transcriptomic landscape at haplotype-specific resolution through RNA-Seq of MHC-homozygous B-LCLs that represent three prominent MHC haplotypes (A2-B46-DR9, A33-B58-DR3, and A1-B8-DR3), and showed that MHC-wide gene expression patterns were distinguished by the underlying MHC haplotype. We were able to circumvent phasing ambiguity concerns that arise from the study of diploid genomes through the use of cell lines that are MHC-homozygous carrying MHC haplotypes that display CEH properties. We identified 36 genes that were differentially expressed in the context of MHC haplotype, and further distinguished haplotype sequence variants that can function as regulatory variants and exert allele-specific gene expression control on these differentially expressed haplotype-specific genes. Via mapping of haplotype sequence variations to B-LCL-specific eQTL that are reported to regulate these differentially expressed genes, we identified correlations between unique allelic combinations of eQTL and expression levels of their target genes. Importantly, these allelic combinations of eQTL are structured in specific MHC haplotype sequences.

A recent study has shown multiple *cis*-acting SNPs, presented as a haplotype, that influence gene expression variability in other parts of the human genome; these haplotypes are comprised of up to four SNP sites at most (Garnier *et al.* 2013). Our study expanded the notion of a haplotypic effect on gene expression within a critically important region of the human genome, which is remarkable for its association with many complex traits and diseases. We demonstrate haplotype-specific allelic combinations within the MHC region, consisting of between 12 and 546 eQTL sites, where these eQTL can be in linkage disequilibrium at distances of >350 kb. Interestingly, we also found allelic combinations of eQTL that not only direct the expression level of >1 gene but also result in divergent expression of the target genes.

An important aspect of this work is the intricate relationship of MHC haplotype sequence variations with eQTL and their subsequent effects on gene expression. We further describe hotspots within the MHC where eQTL are enriched and showed that haplotype-specific eQTL combinations influence the expression of genes in the HLA-A (29.8–30.0 Mb), HLA-C (31.2–31.4 Mb), C4A (31.8–32.2 Mb), and HLA-DRB (32.4–33.0 Mb) regions. This suggests that haplotype sequence variations within or proximate to these regions play an important role in exerting haplotype-specific gene regulatory effects, whereas the expression of genes in other parts of the MHC region are not as tightly controlled by MHC haplotype sequences. One could argue that such observations are due to the high gene density occurring in these hotspot regions; however, we also observed intervals within the MHC region (*e.g.*, 31.4–31.8 Mb; Figure 6B) that are similarly gene-dense, but their expression is not associated with haplotype sequence variation.

By examining the MHC transcriptomic landscape at the haplotype level, we observed that eQTL SNP alleles are segregated by haplotype. There are at least two possible models that accommodate these findings. The first is that the eQTL SNP alleles represent multiple *cis*-acting loci that independently influence gene expression, and that haplotypes combine multiple contributive genes resulting in greater gene expression variation across haplotypes. The second is that the high degree of sequence conservation within particular CEHs and their high frequency in the population skew the results of eQTL studies such that SNPs across a large DNA sequence remain associated with gene expression. Further investigation of the relationship between haplotype and eQTLs are required to inform human genetics approaches within the MHC.

Our data demonstrates that MHC haplotype sequence diversity can impact transcription variability in the MHC region. Our integrated analysis, which relates haplotype sequence variations with eQTL SNPs, has enabled us to differentiate potential functional variants that have a direct effect on gene regulation from sequences that are merely in linkage disequilibrium. Through the analysis of MHC haplotype sequences, our study provides an approach for elucidating the association of MHC haplotypes with different phenotypes and presents a way forward for assessing disease risk related to MHC gene expression.

## Supplementary Material

Supplemental material is available online at www.g3journal.org/lookup/suppl/doi:10.1534/g3.117.043828/-/DC1.
