# Supplementary material for: Unique Allelic eQTL Clusters in Human MHC Haplotypes
Source: G3 (Bethesda). 2017 Jun 9;7(8):2595–604. doi: 10.1534/g3.117.043828 (PMC5555465; doi:10.1534/g3.117.043828)
Supplement: Supplementary file 1 [file 2595FileS1.docx]

**Unique Allelic eQTL Clusters in Human MHC Haplotypes**

Tze Hau Lam, Meixin Shen, Matthew Zirui Tay, Ee Chee Ren

**
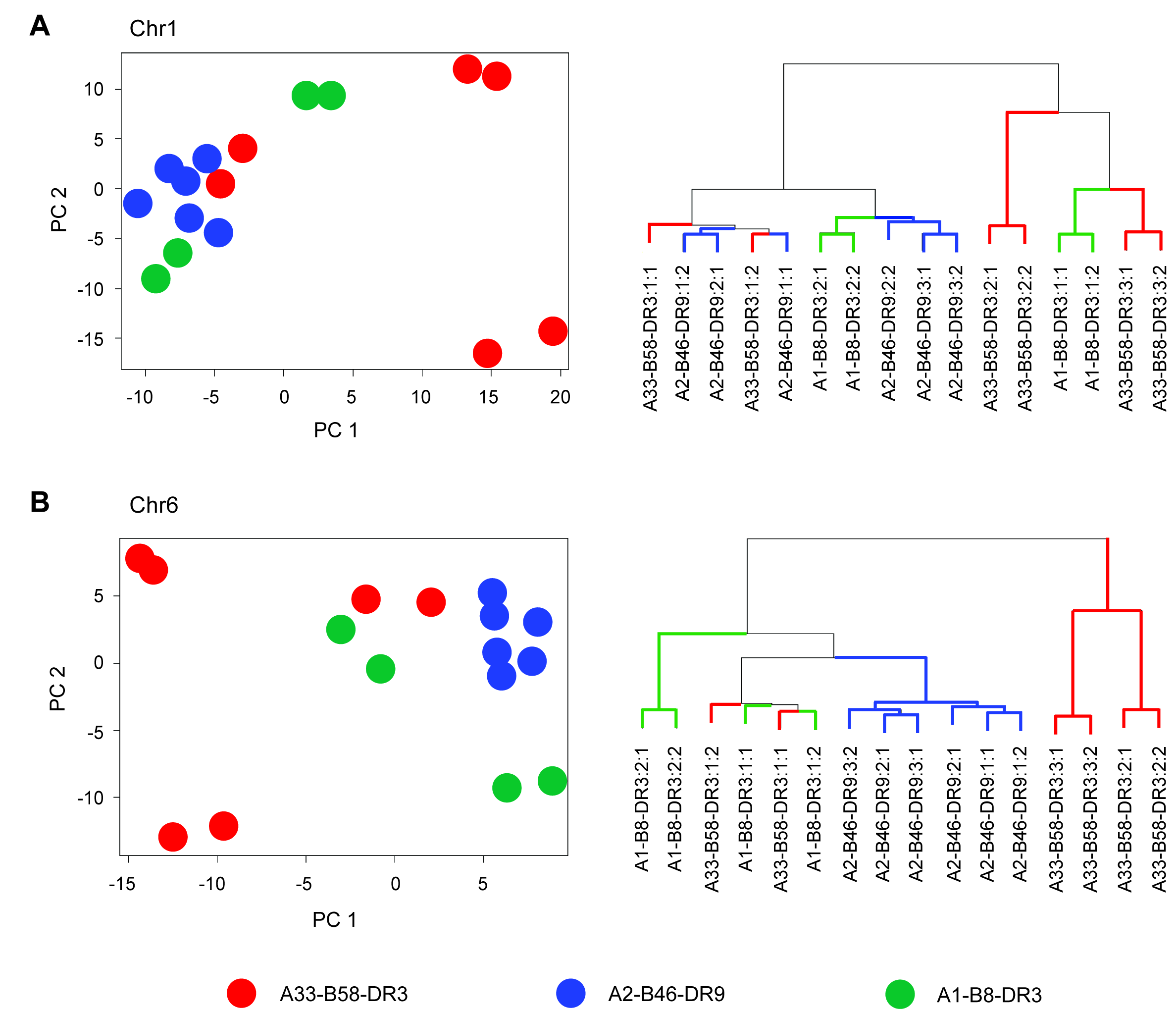
Supplemental Material Figures:**

**Figure S1.** Hierarchical and principal component analysis based on expression of genes in (A) Chromosome 1 (B) Chromosome 6 (excluding genes within MHC). Expression profiles were derived from RNA-sequencing of eight B-LCLs with two libraries generated per B-LCLs. Blue indicates B-LCLs carrying A2-B46-DR9 haplotype; red indicates B-LCLs carrying A33-B58-DR3 haplotype and green indicates B-LCLs carrying A1-B8-DR3 haplotype.

**
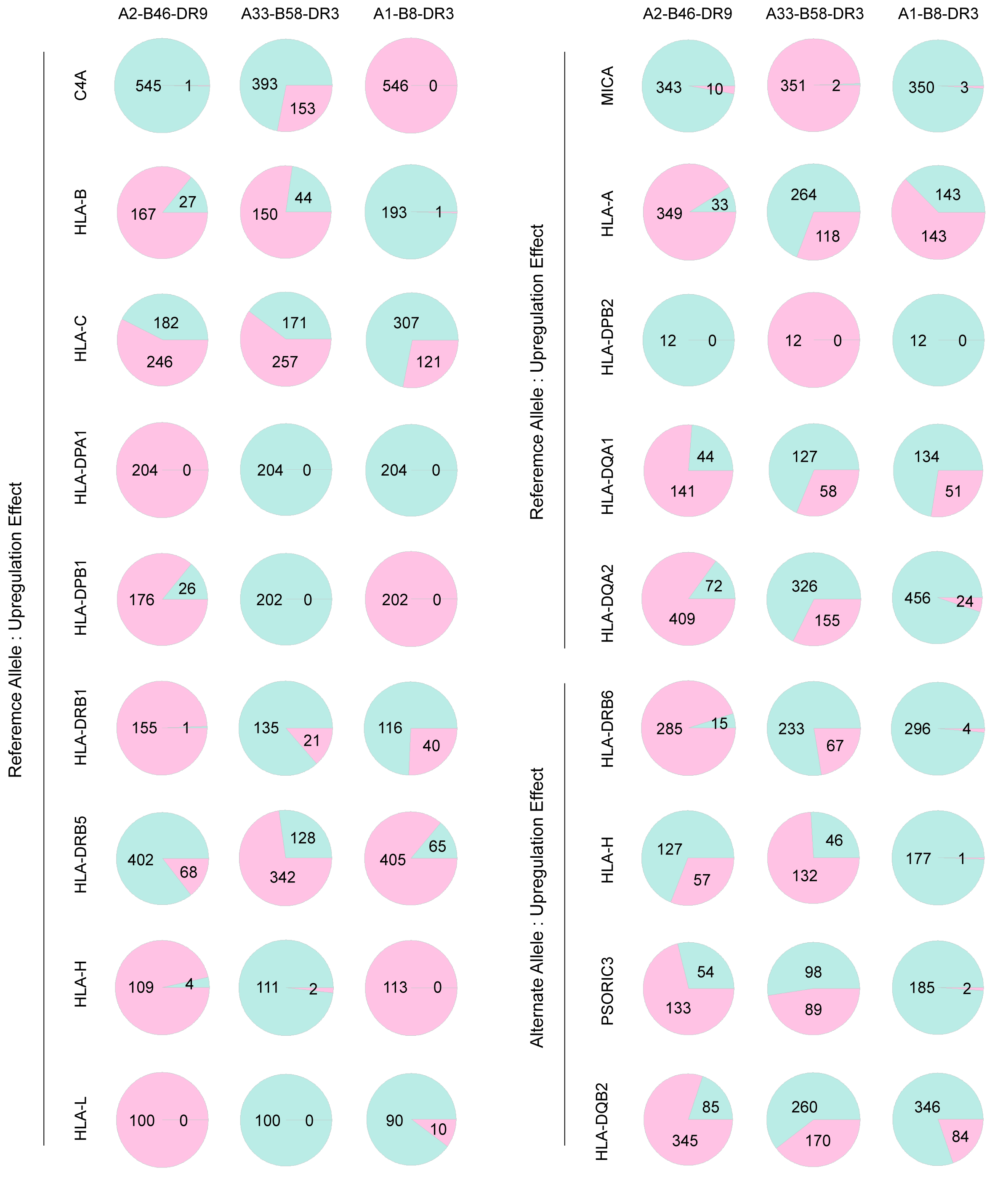
**

**Figure S2.** Allelic proportions of haplotype sequence variations mapping to eQTL with their corresponding genes. These eQTL are retrieved from the GTEx consortium database (Release V6). Blue: eQTL with reference allele. Pink: eQTL with alternate allele.

**Figure S3.**

**
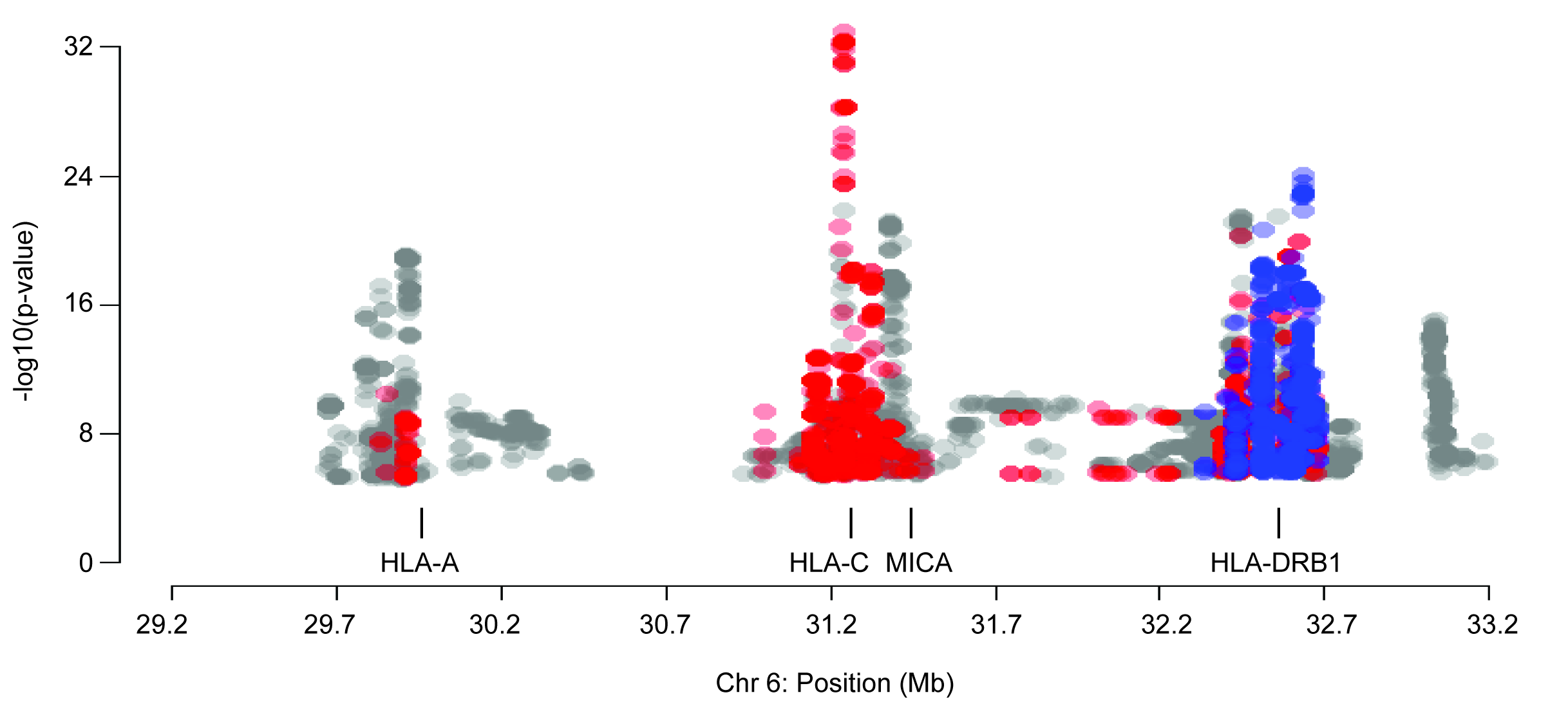
**

**Figure S3.** Genomic position of eQTL associated with the expression of the 17 differentially expressed genes in MHC region. Grey: eQTL affecting the expression of one gene locus. Red: eQTL affecting the expression of 2 gene loci. Blue: eQTL affecting the expression of 3 gene loci.
